# Excipients for Cerium Dioxide Nanoparticle Stabilization in the Perspective of Biomedical Applications

**DOI:** 10.3390/molecules30061210

**Published:** 2025-03-08

**Authors:** Svetlana A. Titova, Maria P. Kruglova, Victor A. Stupin, Natalia E. Manturova, Raghu Ram Achar, Gouri Deshpande, Vladimir A. Parfenov, Ekaterina V. Silina

**Affiliations:** 1 I.M. Sechenov First Moscow State Medical University (Sechenov University), Moscow 119991, Russia; honey.liebe@mail.ru (S.A.T.); marykruglova@live.ru (M.P.K.); vladimirparfenov@mail.ru (V.A.P.); 2 Pirogov Russian National Research Medical University, Moscow 117997, Russia; stvictor@bk.ru (V.A.S.); manturovanatali@yandex.ru (N.E.M.); 3 JSS Academy of Higher Education & Research, Mysuru 570015, Karnataka, India; rracharya@jssuni.edu.in; 4 Regional Institute of Education (RIE NCERT), Mysuru 570006, Karnataka, India; gourideshpande91@gmail.com

**Keywords:** lanthanides, rare earth metals, nanoparticles, nanocerium, cerium oxide, excipients, biopolymers, carboxylic acids, dextran, collagen, chitosan, liposomes

## Abstract

Rare earth metal nanoparticles, some of which are already widely used in medicine, are of growing interest in the modern scientific community. One of the promising rare earth metals for biomedical applications is cerium, specifically its oxide form, which is characterized by a higher level of stability and safety. According to a number of studies, cerium dioxide has a wide range of biological effects (regenerative, antimicrobial, antioxidant, antitumor), which justifies the interest of its potential application in medicine. However, these effects and their intensity vary significantly across a number of studies. Since cerium dioxide was used in these studies, it can be assumed that not only is the chemical formula important, but also the physicochemical parameters of the nanoparticles obtained, and consequently the methods of their synthesis and modification with the use of excipients. In this review, we considered the possibilities of using a number of excipients (polyacrylate, polyvinylpyrrolidone, dextran, hyaluronic acid, chitosan, polycarboxylic acids, lecithin, phosphatidylcholine) in the context of preserving the biological effects of cerium dioxide and its physicochemical properties, as well as the degree of study of these combinations from the point of view of the prospect of creating drugs based on it for biomedical applications.

## 1. Introduction

Nanoparticles of rare earth metals are becoming increasingly popular in the modern world for solving a number of biomedical problems. One of the promising lanthanides is cerium, a metal with variable valence [1], which provides it with antioxidant and enzyme-like properties [2,3]. And its most promising form at the moment is cerium dioxide [4,5], which, according to many studies, has regenerative [5,6,7,8], antimicrobial [9,10,11], and redox activity [12,13,14,15], which determines its prospective application. However, despite the available data and the possible potential of cerium dioxide application in biomedicine, the problem associated with the creation of final dosage forms based on it is still relevant [16]. An interesting fact is that from the chemical point of view we consider one compound—cerium dioxide—but the data of different researchers are represented by a wide variety of effects and different degree of their manifestation. On this basis, we can assume that the important aspects that ensure the realization of the effects of cerium dioxide are the choice of its synthesis method [5,17], and, of course, the choice of the excipient [18].

Excipients, which are commonly understood as components that do not have the properties of a drug substance, for a long time were positioned as completely indifferent substances [19], and their main functional values were mainly attributed to the properties of adjusting the organoleptic properties of preparations [20], giving the optimal dosage form [21], increasing the weight of the dosage form [22], facilitating dosing [23], and providing resistance to adverse environmental factors [24]. Later it was found that many of them have their own pharmacological activity [25], and can reduce [26], and sometimes on the contrary, increase [27,28] the level of toxicity, affecting the rate of release [29] and activity of the main active ingredient [30].

In the case of nanoparticles of rare earth metals, and specifically cerium dioxide, the choice of excipient is a very important problem, because in addition to ensuring the proper levels of effect, stability, it should also have the property of high biocompatibility, and, in the case of lanthanide nanoparticles, also the ability to prevent their aggregation, and thus to provide their nanoscale and ability to pass through biological membranes, which also plays a leading role in the realization of the effectiveness of this group of potential drugs.

The significance of this problem becomes evident when analyzing the international databases PubMed and ScienceDirect. For the PubMed database, searches were performed using the keywords: « “rare earth metals” and “excipients”, “nanoparticles” and “efficiency” and “excipients” and “medicine”, “cerium” and “excipients”, “lanthanides” and “excipients”, “cerium oxide” and “hyaluronic acid”, “cerium oxide” and “polyvinylpyrrolidone”, “cerium oxide” and “chitosan”, “cerium oxide” and “dextran”, “cerium oxide” and “lecithin”, “cerium oxide” and “citrate”, “cerium oxide” and “collagen”, “cerium oxide” and “gelatin”, “cerium oxide” and “polyacrylate”, and “cerium oxide” and “liposome”. For PubMed, the data set was 3, 118, 28, 4, 10, 8, 57, 18, 2, 30, 34, 26, 1, and 2 publications, respectively. According to the inclusion and exclusion criteria, the filters «Clinical Trial», «Meta-Analysis», «Randomized Controlled Trial», «Review», and «Systematic Review» were set. For the categories «cerium» and «excipients», «lanthanides» and «excipients», «cerium oxide» and «hyaluronic acid», «cerium oxide» and «polyvinylpyrrolidone», «cerium oxide» and «chitosan», «cerium oxide» and «dextran», «cerium oxide» and «lecithin», «cerium oxide» and «citrate», «cerium oxide» and «collagen», «cerium oxide» and «gelatin», «cerium oxide» and «polyacrylate», and «cerium oxide» and «liposome», search restrictions were not imposed due to the technical absence of classification of publications on this query in the PubMed system. The final number of publications for analysis was 1, 12, 28, 4, 10, 8, 57, 18, 2, 30, 34, 26, 0, and 0, respectively.

Due to the lack of database specialization in medical, biological and pharmaceutical fields of scientific knowledge, the query for ScienceDirect was adapted by the keywords: “rare earth metals” and “excipients”, “metal-based nanoparticles” and “efficacy” and “excipients” and “medicine”, “cerium” and “excipients” and “medicine”, “lanthanides” and “excipients” and “medicine”, “cerium oxide” and “hyaluronic acid”, “cerium oxide” and “polyvinylpyrrolidone”, “cerium oxide” and “chitosan”, “cerium oxide” and “dextran”, “cerium oxide” and “lecithin”, “cerium oxide” and “citrate”, “cerium oxide” and “collagen”, “cerium oxide” and “gelatine”, “cerium oxide” and “polyacrylate”, and “cerium oxide” and “liposome”. The number of publications was 77, 34, 273, 190, 644, 750, 2432, 713, 192, 1650, 1085, 1028, 175, and 1185, respectively. According to the inclusion and exclusion criteria, the filters “review articles” and “research articles” were applied, resulting in a number of publications of 46, 18, 160, 105, 440, 533, 1598, 471, 112, 1222, 755, 670, 103, and 730, respectively. Additional filters were used to limit the data set to the target field of scientific knowledge: “Biochemistry, Genetics and Molecular Biology”, “Materials Science”, “Pharmacology, Toxicology and Pharmaceutical Science”, “Medicine and Dentistry”, “Immunology and Microbiology”, “Agricultural and Biological Sciences”, and “Neurosciences”. The number of publications according to the filters used was 31, 16, 120, 69, 321, 271, 271, 901, 331, 84, 248, 543, 457, 58, and 532, respectively. There were no restrictions on the date of publication. The total number of publications selected for further analysis was therefore 230 for PubMed and 4253 for ScienceDirect. 

Based on the literature and research data, the main excipients used for cerium dioxide nanoparticles are represented by the following classes of compounds: biopolymers, carboxylic acids and their salts, and lipid derivatives. These compounds can be used both in the process of nanoceria synthesis (e.g., when added prior to synthesis to obtain stable cerium dioxide sols) and added to the final synthesized compound, allowing to preserve its specific physicochemical and biological properties. However, taking into account the fact that the effects of rare earth metal nanoparticles and cerium itself are not sufficiently studied, it is impossible to consider only the assumed results of their interaction with excipients, relying only on the data of physicochemical properties of individual substances. For example, it was shown that during the interaction of cerium dioxide and dextran, new compounds can be formed, which also depended on the ratio of the added excipient to cerium dioxide, and thus the properties of the final product can change [31]. Considering the totality of these factors, the choice of excipient should take into account not only its individual properties, but also its concentration, the moment of addition (before or after nanoparticle synthesis), the possibility of its potential interaction with cerium dioxide, and the final effect to be achieved.

**Figure 1:**
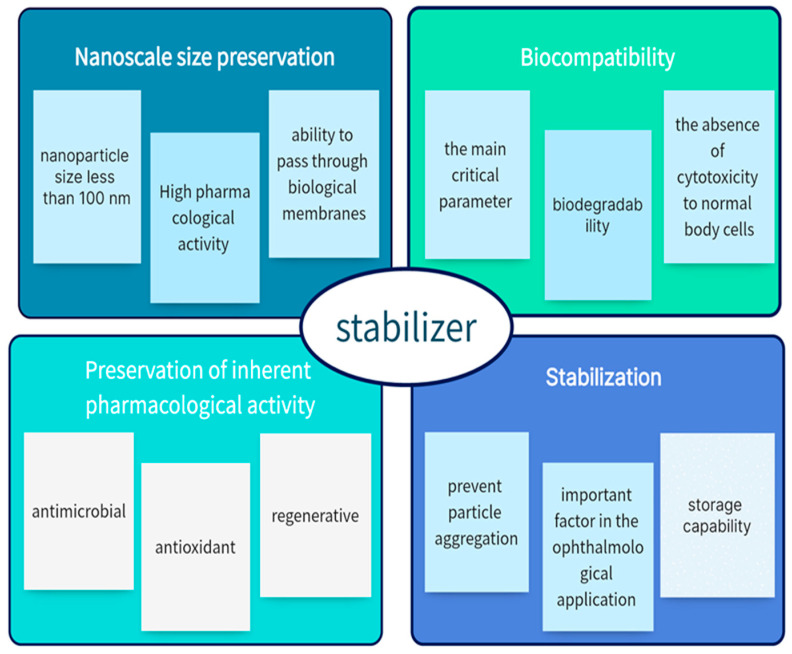
Perfect stabilizer for nanoceria.

Thus, the aim of this review was not only to summarize the available data on the main excipients used for nanoceria, but also on their application technology and their role in the formulation (ratios used, moment of addition to cerium dioxide), as well as the final effects as results of these interactions (Figure 1). 

## 2. Cerium Dioxide Nanoparticles and Biopolymers

The main biopolymers used to stabilize cerium dioxide nanoparticles are hyaluronic acid, dextran, chitosan, polyacrylate, and polyvinylpyrrolidone [32,33]. It has been observed that they can influence the bioavailability values and pharmacological effects of cerium oxide [31,34]. For some biopolymers, such as, for example, polyacrylate, information on these properties is limited to data on cytotoxicity and isolated reports on the intensification of antiviral action of nanoceria [35]. Despite this, cerium dioxide nanoparticles in combination with polyacrylate are characterized by a narrow range of particle sizes. Thus, its particle size ranges from 3.0 ± 0.8 to 9.9 ± 2.5 nm with hydrodynamic diameter from 3.1 ± 0.3 to 5.9 ± 2.9 nm and zeta potential −1.6 ± 0.2 mV [34]. When polyacrylate is added after synthesis, particle size ranges from 1 to 3 nm [35]. Thus, polyacrylate can be characterized as a stabilizer that provides ultra-small and uniform particle size. This may be the reason for the observed antiviral effect. However, the low zeta potential value may indicate low stability during storage. This explains the small number of studies on the combination of nanoceria and polyacrylate.

For others, however, the interactions are very specific. These include polyvinylpyrrolidone, a hydrophilic, biodegradable, and nontoxic biopolymer that has good stabilizing properties and is widely used in medicine [36,37]. In particular, polyvinylpyrrolidone-coated cerium oxide showed antioxidant and cytoprotective effects in brain injury in an in vitro study using neuroblastoma and pro-monocytic U937 cells and in an in vivo study using rats [36,38]. A possible reason for this nanoceria effect is that polyvinylpyrrolidone with a molecular weight of 40 Kilodalton (KDa), added to cerium nitrate hexahydrate before nanoparticle synthesis at a ratio of 0.125 mM:2.5 mM, provides an ultra-small particle size with spherical shape. Polyvinylpyrrolidone-coated cerium oxide had a particle size of 3.49 ± 1.11 nm, which promotes high permeability across the blood–brain barrier. However, it is also characterized by small values of hydrodynamic diameter (6.49 ± 0.56 nm) and zeta potential (5.65 ± 0.42 mV) [36]. It was observed that the antibacterial and regenerative properties of nanoceria were not reduced by this stabilization approach [38,39]. Limited in vitro studies evaluating the toxicity and toxicokinetics of polyvinylpyrrolidone-stabilized cerium nanoparticles have also been reported in the literature. The nanoparticles were synthesized with polyvinylpyrrolidone (PVP) of molecular weight 10 KDa and 40 KDa were added after synthesis. PVP 10 KDa and PVP 40 KDa were taken relative to 4.34 g cerium nitrate hexahydrate in the amount of 0.00005 g and 0.00008 g, respectively. The nanoparticles studied were spherical in shape and 4.3 ± 0.5 nm in size, with a hydrodynamic diameter of 25.9 ± 0.4 nm (PVP 10 KDa) and 32.8 ± 0.5 nm (PVP 40 KDa). The zeta potential was −5.3 ± 1.1 mV (PVP 10 KDa) and −6.8 ± 3.0 mV (40 KDa). They reported no evidence of cytotoxicity and apoptosis in cell cultures, but did observe activation of the transcription factor EB (essential regulator of autophagy), which may have applications in the development of treatments for diseases associated with impaired lysosomal function and may be one of the first signs of specific toxicity of metal nanoparticles [40]. It can be concluded that the nanoparticles produced with PVP 40 KDa had better properties and were more stable than those produced with PVP 10 KDa. The addition of PVP before and after synthesis gave comparable results. Therefore, the selection of the optimal synthesis strategy requires additional toxicity studies.

In contrast to the less studied combinations mentioned above, the so-called «dextran synthesis» is one of the most popular techniques for the preparation of nanoceria due to its high biocompatibility and the relative simplicity of the preparation methodology [31,32,41,42]. In addition to the stabilizing effect itself, the use of dextran enhances the antibacterial effect of cerium oxide nanoparticles, including against antibiotic-resistant *Escherichia coli* (*E. coli*) biofilms [43]. High pharmacological activity of the dextran–nanoceria complex against such microorganisms such as *S. aureus*, *S. epidermidis*, *E. coli*, *E. faecalis*, and *P. aeruginosa* has been reported [31,44,45,46,47]. Notably, the antibacterial properties of nanoceria were maintained when dextran with a wide range of molecular weights (4 to 10 KDa) was used [31,45,47]. It should be noted that the intensity properties of the dextran–nanoceria complex are dose-dependent, and according to several researchers have an optimum at pH = 9 [31,45,48]. An additional factor influencing the antibacterial effect has been identified as the ratio of dextran to cerium dioxide. Thus, in the course of comparative analysis performed with *E. coli*, it was found that the compositions in which the ratio of nanoceria and dextran by weight was 1.0 g:0.5 g and 1.0 g:2.0 g had the highest activity [31]. The high antibacterial effect of the composition of cerium dioxide 1 mL:0.1 M dextran 10 KDa 2 mL was demonstrated by Ece Alpaslan et al. [45]. It is noteworthy that in these experiments the nanoparticle size ranged from 1.2 to 4 nm [31,45]. In summary, studies on the antibacterial properties of dextran-stabilized nanoceria included dextran with molecular masses ranging from 6 to 40 KDa [31,42]. It was added to cerium nitrate prior to synthesis [31,45,46]. The size of the nanoparticles obtained ranged from 0.5 to 4 nm [31,45,46]. The shape of the synthesized nanoparticles was spherical [45]. In the case of prefabricated nanoparticles, triangular and cubic shapes have been described [43]. The hydrodynamic diameter and zeta potential varied considerably. For example, when 0.5 g to 1.0 g of 6 KDa dextran was added to 1.0 g of cerium nitrate, the hydrodynamic diameter was 20 nm and 100 nm, respectively. When the dextran concentration was increased to 3.0 g, the hydrodynamic diameter was up to 200–300 nm [31]. The zeta potential varied from 8.75 ± 4.17 mV to 11.76 ± 3.54 mV when changing from pH = 6 to pH = 9 [45]. 

According to the results of in vitro experiments by Kim S. J. and Chung B. H., the redox properties of nanoceria also depend on the acidity of the medium [49]. On the one hand, this imposes certain limitations on the use of cerium oxide as an antioxidant, but on the other hand, it opens up a prospective use of cerium oxide as an antitumor agent, since cells of malignant neoplasms have a slightly acidic pH [50]. Under in vitro conditions, dextran-coated nanoceria in a wide range of sizes (from 3 to 10.18 nm) had a highly selective cytotoxic effect on cultures of osteosarcoma, A375 melanoma, and neuroblastoma cells; it did not protect lung carcinoma (A-549) and breast carcinoma (BT-474) cells from oxidative stress, while normal cell cultures remained intact [51,52,53]. A comparative analysis of the antitumor effect of cerium oxide and cerium oxide coated with dextran was performed by Miletić M. et al. using the cervical cancer cell line HeLa as an example. The latter had a more pronounced cytostatic effect in vitro [54]. The observed phenomenon is probably due to the combination of functional groups on the cerium surface as well as its own redox properties [55,56]. The antitumor effect was also dose-dependent when the dextran concentration was increased from 0.01 to 0.1 M [32]. When analyzing the synthesis procedure of the described nanoparticles, some peculiarities can be identified. Cerium nitrate was used as a precursor [51,53]. The ratio of cerium nitrate hexahydrate to 1 KDa dextran was 0.125 g and 0.05 g, and 0.250 g and 0.100 g in the two experiments, respectively [53]. The antitumor effect was observed both when dextran was added before [51] and after synthesis [54]. The particle size was 3 to 4 nm (hydrodynamic diameter 40–85 nm) when dextran was added before synthesis [51] and about 3.5 nm (hydrodynamic diameter about 47 nm) when added after synthesis [54]. When the prefabricated nanoparticles were used, the size was 9.52 ± 0.66 nm and the hydrodynamic diameter was 93.17 ± 5.10 nm [52]. Meanwhile, the best zeta potential was observed for the nanoparticles to which dextran was added after synthesis (−19.5 mV). [53].

An alternative biocompatible polymer candidate for the role of excipient for nanoceria is hyaluronic acid. In addition to stabilizing cerium oxide, it has many additional functions. In particular, its compositions have anti-atherosclerotic and anti-inflammatory effects associated with the ability to bind to CD44 receptors of cells [57,58,59]. Further comparison of nanoceria + hyaluronic acid composition, free nanoceria and its complex with dextran by Wang S. et al. demonstrated greater antiatherosclerotic efficacy of hyaluronic acid with a molecular mass of 6 KDa, added after synthesis. The nanoparticles were spherical, with a size of 3 nm, a hydrodynamic diameter of 25 nm, and a zeta potential of −21.78 mV [57]. However, the studies were only carried out in vitro on human fibroblast cell cultures, so it is currently difficult to assess the extent of the prospects for the use of this organometallic complex and its biocompatibility [57].

At the same time, the anti-inflammatory effect of nanoceria with hyaluronic acid has been demonstrated independently of the causes of pathological development (from the model of radial tissue damage to osteoarthritis) and was often accompanied by a regenerative effect [60,61,62]. In addition to the beneficial effects on cells, several studies indicate the ability of the combination of hyaluronic acid and nanoceria to improve the function of ischemic organs [63,64,65] as well as to modulate the microenvironment [66]. These effects have been explained by the antioxidant activity of hyaluronic acid-coated nanoceria [59,62,65]. Hyaluronic acid with a molecular weight between 90 and 172 KDa was added after the synthesis of the nanoparticles [59,65]. Cerium nitrate was used as a precursor [62]. The shape of the particles has been described as spherical [65], cubic [62], and rhombohedral [59]. The particle size ranged from 4.15 nm [65] to 7 nm [59] and the hydrodynamic diameter was about 100 nm [65]. The zeta potential varied considerably. Values from 4 to 25 mV have been reported [59,65].

With regard to malignant neoplasms, the role of hyaluronic acid-coated nanoceria compositions has been described in the context of the induction of apoptosis of triple-negative breast cancer cells and as a means of enhancing the efficacy of photodynamic therapy and photothermal therapy [32,67,68,69]. Nanoparticles provided greater penetration depth of the second near infrared (NIR-II) light and also showed synergy with sonodynamic therapy. Hyaluronic acid increased the targeting of the therapeutic effect to the tumor [67]. These directions, as estimated by Zeng L. et al. from 2021, are named as one of the main vectors for the development of effective tumor treatment [70]. The role of hyaluronic acid in the application of this composition in oncology is to provide a targeting effect on tumor tissue [71].

The antibacterial properties of the combination of nanoceria and hyaluronic acid are considered with the additional introduction of zinc into the organometallic complex of cerium and hyaluronic acid, thus providing an enhancement of the antibacterial properties due to the synergism of metallic nanoparticles [72]. When studied on a wound surface, the role of hyaluronic acid in this case was to enhance the healing of lesions. The differences from the control group were statistically significant. At the same time, the authors of the paper point out the need to balance the targeted effect and the independent toxicity of nanoparticles, which can be achieved by coating the surface with high molecular weight (170 KDa) hyaluronic acid at approximately 10% [72]. Undoubtedly, further research is needed as the use of nanoceria may become one of the possible ways to overcome antibiotic resistance [31].

One of the most extensively studied composites are those of cerium oxide nanoparticles with chitosan. They are recognized as biocompatible, have a homogeneous structure and the particle size remains consistently smaller compared to other biopolymers [32,73,74]. According to Fahmy H.M. et al., this is particularly important for nanoparticles as it is directly related to the risk of toxic effects [75]. The good solubility of this combination, as well as its ability to imbue the surface of cerium oxide with a positive charge, should be noted [32,64,76]. It is believed that this approach reduces potential toxicity and also provides good adhesion to mucosal tissues [77,78].

Recent publications provide a considerable amount of information on a wide range of antibacterial activities, including Methicillin-resistant *Staphylococcus aureus* (MRSA) [39,76,79,80]. Only for nanoceria with a diameter of 60–70 nm, combined with chitosan at a ratio of 0.5 g:1 g, results on the presence of high fungicidal activity against *Aspergillus aureus* and *Agaricus volvaceus* have been reported [81,82]. The effects described, together with the regenerative properties of cerium oxide, are a promising direction for the development of diabetic wound therapy and bone tissue engineering, some of the most labor-intensive areas of regenerative medicine [83,84,85,86,87,88]. The use of chitosan with a molecular weight of 50–190 KDa in relation to cerium oxide 0.100 g:0.0075 g reveals important factors complementary to the pharmacological properties described, namely: the combined prolonged release of cerium oxide (48 h), the ease of application in the form of a gel, or the possibility of exploitation in the form of a medical device (dressing) [85,89,90,91,92]. In all these applications, an additional cytoprotective effect is realized due to the antioxidant effect [93]. The preservation of this property of cerium when coated with chitosan also gives the possibility of using the composition in spinal cord injuries, thus realizing a neuroprotective effect [94,95,96,97]. In summary, studies on the antibacterial and regenerative effects of chitosan-stabilized nanoceria have used cerium nitrate as a precursor [78,79,83,97]. Less frequently, prefabricated nanoparticles have been used [90]. Chitosan with a molecular weight of 50–190 KDa was introduced after synthesis [83]. The shape of the nanoparticles obtained was spherical [79,83], or less frequently cubic [78]. The particle size was generally between 3.61 nm and 50 nm [79,90], the hydrodynamic diameter was up to 174 nm [77], but the zeta potential was only between 0.26 mV and 9.6 mV [77,83]. 

Interestingly, of the most studied interactions of cerium oxide with excipients, only the chitosan–nanocerium composition remains extremely limited in terms of oncology. The targeting of retinoblastoma cells has been described in the literature, presumably to reduce the risk of development and severity of systemic side effects, as well as to reduce tumor resistance to existing therapeutic options [98,99,100,101]. In contrast, attempts to combine cerium and chitosan nanoparticles with antimetabolite drugs under in vitro conditions failed to demonstrate an increase in efficacy compared to samples containing only fluorouracil and chitosan supplemented with silver nanoparticles [102].

In contrast, a considerable amount of data addresses the question of the potential use of nanoceria chitosan in ophthalmology [103]. The potential use in the therapy of age-related macular dystrophy has been described due to protection against apoptosis, decreased production of anti-inflammatory cytokines, reduction of oxidative stress, and several other factors [64,104,105,106,107,108,109]. It is believed that the 42–43-fold increase and acceleration of permeability for cerium oxide is achieved precisely by adding chitosan to the composition [110,111,112,113]. The antioxidant properties of nanoceria reduce the severity of dry eye syndrome under experimental conditions by increasing the activity of bocaloid cells [114,115]. An improvement in the morphological characteristics of conjunctival and corneal cells has been observed in vitro and in a mouse model [111,115,116]. An important aspect is the preservation of the antioxidant effect when the standard nano-size limits are exceeded by chitosan-stabilized cerium oxide particles at 100 nm [115]. The biocompatibility of the composition with chitosan of molecular mass 50–190 KDa was evaluated on the retinal pigment epithelial cell line ARPE-19. As a result, no signs of inflammatory reactions were found, emphasizing the promising application of chitosan-containing formulations [117]. The introduction of an additional pharmaceutically active substance (pilocarpine) into the nanoceria–chitosan complex makes it possible to extend the range of ophthalmological applications while maintaining the potentiating effect on the permeability of the complex through the cornea. It is claimed that the bioavailability of pilocarpine under in vivo conditions is increased 250-fold [111,112,113]. Thus, such multifunctional systems can provide biocompatibility, reduce oxidative stress and reduce the effects of inflammatory factors [118]. Compositions containing nanocerium, chitosan, and alginate have also been developed. The role of cerium was to impart an antibacterial effect to the membranes. The organic component provided elasticity of the products and stability during deformation [119]. In the studies describing these effects, the synthesis process had a number of peculiarities. Thus, unlike the previously discussed methods, cerium chloride was used as a precursor. Chitosan was added before the synthesis of the nanoparticles [108,115]. The resulting particles had a spherical shape [115]. The particle size ranged from 5 nm to 100 nm and a zeta potential of 40.9 ± 3.6 mV was reported [108,115]. These characteristics can be explained by the fact that stability is of paramount importance in the ophthalmic application of drugs, confirming the importance of the zeta potential.

Hydrophilic biopolymers such as collagen and gelatin play a special role in the preparation of cerium-containing pharmaceutical compositions [120,121,122]. Due to their high biocompatibility [95], optimal rheological [123], stabilizing properties [39], mucoadhesion, and high affinity to the tissues of the wound surface, they have found application in a wide range of directions in the development of agents for use in medicine [124,125,126]. In particular, optimal levels of mechanical strength and porosity have led to the development of agents for dentistry and bone tissue engineering [84,126]. According to in vitro and in vivo studies, collagen scaffolds promote accelerated tissue regeneration and differentiation in the injured area [84,126]. According to Chen X. et al., it is a response to a specific stimulus: the generation of reactive oxygen species. Synergism of antioxidant effect of nanoceria and biopolymer matrix was observed [127]. In the study with ovarian cancer cells, cerium nitrate was used as a precursor and collagen was added after synthesis. It was found that this combination, with a cerium oxide particle size of 32.8 ± 4.2 nm, could be a candidate for the role of an antitumor drug [128]. In the study by Zubari W. et al., it was found that the replacement of cerium oxide by its peroxide resulted in an intensification of angiogenesis processes to improve the healing of chronic wounds [129]. As a result of Inbasekar C. and Fathima N. N.’s experiment with collagen fibers obtained ex vivo, not only does the biopolymer have a stabilizing effect on nanocerium [130], but cerium dioxide also increases the stability of collagen at the molecular level [131].

At the same time, a partial hydrolysate of collagen, gelatin, has become much more popular in biomedicine. Like its predecessor, gelatin with cerium oxide has been considered as a gel scaffold in bone engineering [84] and dentistry [132] with pronounced regenerative properties [133]. According to Bhushan S. et al., the specific antioxidant and antibacterial properties of cerium oxide were retained and the proliferative effect on bone tissue was demonstrated under in vitro conditions on cell culture and in ovo [84]. These results are supported by in vivo studies performed on rats [134]. Xuerui Chen et al. and Jain A. et al. mentioned that the combination of gelatin and nanoceria has antihypertrophic properties for cardiomyocytes [135,136]. Regarding the wound surface, the composition under consideration demonstrated regenerative and antioxidant properties [32,137] on mouse 3T3-L1 fibroblasts and HaCaT human epidermal keratinocyte cell cultures [138,139], as well as in vivo [140,141], even in the presence of concomitant pathology, as shown in several studies on HaCaT human epidermal keratinocytes, the murine leukemia macrophage cell line RAW264.7 cultures and an in vivo model of diabetes [142,143,144]. Evidence of a favorable antibacterial efficacy profile is currently reported for a significant spectrum of microorganisms such as *Pseudomonas aeruginosa* [145,146], *S. aureus*, and *E. coli* [147]. It should be noted that gelatin helps to increase the bioavailability and efficacy of nanoparticles and provides a prolonged and uniform release, which may lead to better tolerability in the long term [148,149,150]. A much smaller number of studies have looked at the use of the combination of gelatin and nanoceria in other areas of medicine. A number of works provide data on the possibility of using this combination as an antioxidant and regenerative agent for the stimulation and regeneration of neurons [64,151,152] in cell cultures and in vivo [153], anti-inflammatory [154], including in lesions of the central nervous system [155], as well as in cardiology [76] and ophthalmology [156]. As a more promising excipient, it is worth considering gelatin type A, which achieves a smaller particle size than type B (20 nm and 43 ± 5 nm, respectively) [136,139,151]. From a technological point of view, the use of gelatin as a nanoparticle stabilizer can be characterized as follows. In most cases, gelatin was added after synthesis [84,133,134,144]. Both cerium nitrate [84,133,134] and cerium chloride [136,155] were used as precursors. The particle shape was predominantly spherical [84,133,153], but rhombohedral has also been described [139]. The particle size ranged from 2.5 nm to 80 nm [140,155]. The hydrodynamic diameter ranged from 20 to 195 nm. The zeta potential of the particles ranged from −12.35 ± 1.39 mV to −50 mV [133,155]. Thus, it can be concluded that the choice of the optimal synthesis strategy using gelatin requires further studies to achieve the best zeta potential value. 

In summary, it should be noted that numerous studies describe the interaction of cerium oxide and biopolymers. Their use is widespread, but none of them can be characterized only as a stabilizer, since biopolymers themselves are capable of producing additional effects, as well as influencing the efficiency and spectrum of action of cerium dioxide. The main results of studies of interaction between cerium dioxide nanoparticles and biopolymers are summarized in Table 1.

## 3. Cerium Dioxide Nanoparticles and Carboxylic Acid Derivatives

The great popularity of the use of carboxylic acids and their derivatives as stabilizers for cerium oxide nanoparticles is due to a combination of reasons. Firstly, as mentioned above, the pharmacological effect of nanoceria is best realized at a slightly acidic pH. Another important factor is the fact that the presence of three or more carboxyl functional groups ensures the aggregation stability of the particles, contributes to the maintenance of the biological effects of cerium, and serves as an additional source of energy for ATP synthesis. [17]. Such stabilizers include mellitic [157] and aconitic acids [158], but the biological effects and the possibility of using their compounds with cerium in medicine are not currently considered in the literature. A larger amount of data is available for L-amino acids. For cerium oxide synthesized with glycine, proline, valine, histidine, cysteine, and glutamic acid, in vitro studies have been carried out and the results showed high stability as well as the possibility of regulating the morphology of the nanoparticles [159,160]. For cysteine and glutamic acid, an evaluation of their properties in the context of biomedicine has been carried out. In particular, David Schubert et al. concluded that cerium oxide nanoparticles reduce oxidative stress induced by glutamic acid ingestion in HT22 nerve cell culture [161]. In turn, derivatives of cysteine and cysteine with glutamic acid (acetylcysteine and cysteine–arginine–glutamic acid–lysine–alanine peptide) demonstrated antioxidant effects [162] and targeting effects on tumor tissue [163]. An antitumor effect has also been found for acetic acid-stabilized cerium oxide. This was demonstrated in a study using human colorectal cancer cells HT-29 and the human fetal foreskin fibroblast cell line HFFF2 [164]. There is currently no data on experiments to evaluate the realization of other pharmacological effects of nanoceria. At the same time, 2024 publications indicate a renewed interest from the international scientific community in the application of carboxylic acids. A large-scale in vitro study of compounds with sixteen organic acids on the stability of cerium oxide nanoparticles showed that nanoceria stabilized by citric, malic and isocitric acids had the highest aggregation stability (particle size 4.2 ± 1.2 nm) [165]. Malic acid was also shown to have high antibacterial activity against *E. coli* and *S. aureus*, including reduction of biofilm formation [166].

The most studied stabilizer of cerium dioxide nanoparticles for medical applications is citric acid and its salts, which are highly biocompatible [167,168,169]. The addition of citrate makes it possible to achieve an optimal particle size (in the range of 1 to 7 nm) and also contributes to an increase in the permeability of cerium oxide through cell membranes due to the negative zeta potential, resulting in an enhancement of the antioxidant effect and a significant reduction in toxicity [170,171,172,173]. A comparative analysis of the cellular uptake of polymer and citric acid stabilized nanoceria showed the greater efficiency of the latter [174,175]. It should be noted that there is no universal way to realize the regenerative properties of cerium oxide + citrate in every phase of wound healing: the biological activity can have opposite effects depending on the synthesis method and the nanoparticle concentration used. At the same time, the use of cerium dioxide and citrate in a molar ratio of 1:1 has been found to be relatively universal in the development of agents with a regenerative effect [17]. It is worth noting that citrate-stabilized nanoceria can be incorporated into polymeric pharmaceutical compositions by incorporating them into a hydrogel matrix or microspheres, preserving the antioxidant and regenerative effects [73,176,177]. The results of experiments on the antibacterial activity of citrate-stabilized cerium dioxide are currently contradictory. In an extensive study with six bacterial strains and two fungal strains, a dose-dependent antimicrobial effect was found, which was most significant for *E. coli* [178]. Another paper from 1999 reported the low ability of citrate nanoceria to exert a bacteriostatic or bactericidal effect [179]. This phenomenon can be explained by the fact that standard methods for assessing antimicrobial activity are not relevant for cerium oxide nanoparticles. Another way to solve the problem may be the correct choice of doses (in particular, 10^−3^ M with a cerium:citrate ratio of 1.086 g:0.48 g was found to be the optimal concentration), as well as the combination with polymeric carriers to enhance antibacterial activity and blood–brain barrier penetration [178,180]. Pharmacokinetic parameters (especially distribution) may also be dose-dependent and correlate with the route of administration [181].

The unique spectrum of pharmacological effects observed for citrate-stabilized cerium dioxide is of particular interest. The current literature provides data on immunomodulatory and antiviral effects [35], prophylactic effects in sunburn [182], and therapeutic effects in multiple sclerosis [183,184,185], as well as reproductive disorders in males [186] and many other pathologies [98]. As a possible reason for this phenomenon, it can be assumed that the best stabilizing properties of citrate (the maximum described particle size of citrate-stabilized nanoceria is no more than 63 ± 15.25 nm, regardless of the moment of addition of the excipient, indicating minimal aggregation) contribute to a fuller realization of the potential of these nanoparticles [183]. At the same time, contradictory data on the pro-oxidant, cytotoxic effect of citrate-stabilized cerium nanoparticles with a diameter of 5 nm, obtained by a hydrothermal method with a molar ratio of cerium chloride and citric acid of 1:1, on the brain and liver parenchyma are reported in the literature, which requires further investigation of the safety profile [167,187,188,189].

When analyzing the synthesis strategy of citrate-stabilized nanoceria, several peculiarities can be observed. Although cerium nitrate is used as a precursor [17,178,179], the use of cerium chloride is mentioned much more frequently than when working with polymers [170,180,186]. The addition of citrate before [170,186,187] and after synthesis [167,183,188] is about equally common. However, the addition of citrate before synthesis results in smaller nanoparticle sizes, specifically from 2 [186] to 5 nm [180,187] (hydrodynamic diameter from 4.9 [186] to 130 nm [17]). The morphology of the particles was nearly isotropic [170,186]. In comparison, when citrate was added after synthesis, the particle size was less uniform and ranged from 1 [171] to 31.2 nm [167] (hydrodynamic diameter from 2.9 [183] to 200 nm [167]). Zeta potential values were comparable. When added before synthesis, they ranged from −20 mV to −53 ± 7 mV [170,187], and when added after synthesis from −23 mV to −56 ± 8 mV [167,183]. No clear relationship was found between the synthesis technique and the effects reported in the literature. The main results of the studies on the interaction between cerium dioxide nanoparticles and carboxylic acid derivatives are summarized in Table 2.

## 4. Cerium Dioxide Nanoparticles and Liposomes

Like other rare earth metals, cerium nanoparticles have a high affinity for lipid compounds. This would be expected to provide an opportunity for the production of liposomal forms of cerium nanoparticles. Such an approach has been investigated by coating the surface of cerium nanoparticles with surfactant (composed mainly of lipids, among which phosphatidylcholine and lecithin predominate) [190,191,192]. According to the researchers, such a coating promotes endocytosis of the drug. At the same time, a limitation was identified: the risk of aggregation of nanoparticles with proteins and lipids in the alveoli and, as a consequence, the risk of developing lung function disorders [192]. It should be noted that the studies were performed using computer modeling, which introduces additional nuances when extrapolating the data to a real organism. The issue of interaction between nanoceria and lecithin, a surface-active phospholipid that is part of the cell membranes of all living organisms, has been addressed in more detail in the literature. Data from in vitro studies of this composition were quite contradictory: no signs of cytotoxicity were found, but the antioxidant properties of cerium were not manifested either [193]. Opposite results with respect to free radicals were obtained in betaTC-tet insulinoma cells, as well as with respect to cytotoxicity in an in vivo study with nanoceria particle size of 5–6.5 nm [188,194]. At the same time, lecithin has its own antioxidant properties [195]. It is worth noting that cerium also has an effect on lecithin, promoting its transformation into an organogel [196]. The combination of lecithin nanoliposomes and gel showed synergistic antioxidant and anti-inflammatory effects when applied as a transdermal therapeutic system containing particles with a size of 5.82 ± 0.24 nm [197]. Therefore, it can be concluded that the interaction between cerium and lecithin is difficult to predict and ambiguous.

Separately the combination of cerium and phosphatidylcholine was also considered, highlighting another potential problem for the embodiment of a technological solution: according to the results, cerium IV causes hydrolysis of phosphatidylcholine and other phosphoric acid esters at both acidic and slightly alkaline pH values. On the other hand, this observation may provide a foundation for the development of treatments for lysosomal accumulation diseases [198,199].

Thus, working with cerium poses additional problems to researchers that do not arise when working with other nanoparticles, including other lanthanides: preservation of the full range of its pharmacological effects, limitation of routes of administration due to the risk of adverse reactions in contact with surfactants, and difficulties in selecting the composition to create biocompatible liposomal forms with satisfactory performance and stability. The main results of studies on the interaction between cerium dioxide nanoparticles and lipid substances are summarized in Table 3.

In summary, it should be noted that the original studies are mainly in vitro studies, which makes it difficult to draw conclusions about the behavior of the pharmaceutical compositions under consideration in the human body. The interaction between nanoparticles (including cerium oxide nanoparticles) and excipients has not yet been investigated and is only indirectly addressed.

## 5. Discussion

In this work, we did not consider the intrinsic pharmacological activity of cerium, but we considered the final set of possible effects when adding different excipients. In analyzing the literature data from a number of studies, we focused on the requirements of excipients and their significant effects when interacting with cerium dioxide, namely:

(1) Biocompatibility: For metallic nanoparticles, the most critical parameter is the absence of cytotoxicity to normal body cells.

(2) Preservation of nanoscale size: For this purpose, various stabilizers are used that are added before or after synthesis to prevent particle aggregation.

(3) Preservation of inherent pharmacological activity of nanoparticles.

Various biopolymers as well as citric acid and its salts meet all these requirements to a greater or lesser extent. The choice of the optimal excipient from this range of compounds may vary depending on the intended use and the dosage form. In terms of biocompatibility, most of the excipients reviewed meet these requirements and have long been used in other areas of medicine. However, there is evidence that toxic effects may be observed when, for example, citric acid and polyacrylate are used to stabilize nanoceria, which should be taken into account [34,175].

Another important task is to maintain the nanoscale size of the cerium dioxide to ensure its ability to penetrate biological membranes and thus produce effects. Considering the data of the publications studied, the range of particle size for each excipient varied greatly, depending on the method of synthesis and the choice of molecular weight of the excipient. Thus, according to the results of the data we studied, the smallest size and size range were obtained when the excipient was added prior to synthesis [17,31,57,178].

The retention of pharmacological activity of ceria nanoparticles is also likely to depend on the amount of excipient added. A lack of excipient may lead to aggregation and formation of larger ceria particles, and its excess may completely cover the nanoparticles and reduce their efficacy or even completely block their effects [31,45].

Among the excipients considered in this work, dextran with a molecular mass of up to 10 KDa is currently considered the most studied and promising [41,45,51,55]. The sizes of the synthesized particles ranged from 1.2 to 304 nm, depending on the synthesis method [31,43]. However, as mentioned above, the optimal way to introduce this stabilizer from the point of view of efficiency of the final composition is to add dextran before the synthesis process [31,41,42,45,46,47,49,51]. There are enough studies in the literature describing the preservation of the main therapeutic properties of cerium dioxide, especially antioxidant [41,43,44,49,51], antibacterial [31,45,46,47], and regenerative [31,44]. The biocompatibility and efficacy of dextran stabilization is also unquestioned based on a significant number of studies in various cell cultures [31,41,42,44,49,51].

The second most studied and potential candidate is chitosan with a molecular weight of 50–150 KDa [83,90,108], which allows the synthesis of particles from 3 to 120 nm [77,83]. It is biocompatible [73,74,77,84,87,90,108], has its own antibacterial properties and retains all the main effects of cerium dioxide [78,79,81,83,84,85,86,90,97,108,115]. However, in contrast to dextran, there is insufficient data on its ability to exert a selective cytotoxic effect on tumor cells and to provide prolonged release.

Information on the interaction of other excipients with cerium dioxide is rather limited. In particular, hyaluronic acid in the molecular weight range from 6 to 172 KDa may be the optimal solution to the problem of realizing the antioxidant effect of nanoceria [57,59,65] with a range of particle sizes from 3 to 250 nm [66,70]. Citric acid, despite the great popularity of its use as a stabilizer, due to the smallest range of nanoparticle sizes (from 1 to 14 nm [182] and from 2 to 30 nm [17,167] when added before and after synthesis, respectively), shows very controversial biological effects (both toxic [175] and beneficial [175,179,187,188]) in vivo, which creates a primary need for a comprehensive and multistep evaluation of the acute and chronic toxicity of citrate-stabilized cerium dioxide.

The biological interaction of nanoceria compounds with other excipients such as polyacrylate [34,35], polyvinylpyrrolidone [36,37], phosphatidylcholine [194], lecithin [193,197], etc. is currently insufficiently studied and does not allow us to draw a reasonable conclusion about the safety and efficacy of these pharmaceutical compositions. 

## 6. Conclusions

In connection with the above, it can be concluded that the development of specific forms of drugs and products for medical use requires careful selection of excipients and a complete step-by-step study of them under in vitro, ex vivo, and in vivo conditions. Currently, the most studied and safe excipients are biopolymers, especially dextran and chitosan. According to the results of the analysis, they allow all the specific biological effects known for cerium dioxide to be maintained, do not affect the physicochemical properties of the nanoparticle, and have a satisfactory safety profile. The possibility of using other excipients requires additional studies.

## Figures and Tables

**Table 1:** Result of interaction of biopolymers and nanoceria («-» means there are no data).

Excipient	Adding Excipient Before/After Synthesis CeO_2_	Morphology	Nanoparticle Size, nm	Hydrodynamic Diameter, nm	Zeta Potential, mV	Effects	Methods	Sources
Polyacrylate	Nanoparticles were not synthesized	-	From 3.0 ± 0.8 to 9.9 ± 2.5	from 3.1 ± 0.3 to 5.9 ± 2.9	−1.6 ± 0.2	Growth inhibition	In vitro *Chlamydomonas reinhardtii*	[34]
Polyacrylate	After synthesis	-	From 1 to 3	-	-	Antiviral	In vitro. L929, EPT and Vero cells	[35]
Polyvinylpyrrolidone	Before synthesis	spherical	3.49 ± 1.11	6.49 ± 0.56	5.65 ± 0.42	Antioxidant	U937 cell line, in vivo	[36]
Polyvinylpyrrolidone	After synthesis	spherical	20 ± 10	-	−32.9	Negative impact on the growth and development of larvae	In vitro, Drosophila melanogaster	[37]
		spherical	4.3 ± 0.5	25.9 ± 0.4; 32.8± 0.5	−5.3 ± 1.1; −6.8 ± 3.0	Induce upregulation of genes of the lysosome–autophagy system	HeLa, TFEB	[40]
Dextran	Before synthesis	spherical	0.5–4	10–100; 110–300	-	High aggregative stability Antimicrobial Regenerative	*E. coli* Human fibroblast culture (BJTERT line)	[31]
		-	2.7–9	-	-	Antioxidant	MIN6 beta cells	[41]
		spherical	3–4	15.5–24	−8.75 ± 4.17, 11.76 ± 3.54	Antimicrobial	*P. aeruginosa*, *S. epidermidis*	[45]
		-	3–4	14	−2	Antimicrobial	*P. aeruginosa*	[46]
		-	-	-	-	Antimicrobial	*E.* *f* *aecalis*	[47]
		spherical	3–4	40–85	0.58 +/− 2.27, 16.68, 17.52, 6.32	Cytotoxicity to tumor cells	Osteosarcoma cells MG-63	[51]
Dextran	After synthesis	-	-	-	-	Antioxidant	In vitro	[49]
		-	5.2	15–72	-	Cytotoxicity to tumor cells	neuroblastoma cell lines SK-N-AS, SMS-KAN, LA-N-6 and IMR-32. SK-N-AS	[53]
		-	3.5	47	-	Cytotoxicity to tumor cells	The human cervical cancer cells HeLa	[54]
		cubic	3.0 ± 0.63	-	-	Less absorption by cells compared to other stabilizers	The human gastric cancer cells BGC-803	[55]
		-	From 5–8 to 2 500	-	-	Photosensitivity	normal cell line HUVEC, skin cancer cell line CCL-30	[56]
Dextran	Nanoparticles were not synthesized	cubic and triangular	277 ± 27	-	−25	High aggregative stability, Antimicrobial	In vitro, *E. coli*	[43]
	Nanoparticles were not synthesized	cubic and triangular	9.52 ± 0.66	93.17 ± 5.10	−10.6 ± 1.3	Cytotoxicity to tumor cells	A549, HCT116, Hep3B, Caco-2, HeLa cells	[52]
Hyaluronic acid	Before synthesis	spherical	3	25	−21.78	Anti-atherosclerotic. Antioxidant	In vivo, Mouse aortic smooth muscle cells MOVAS and the RAW 264.7 mouse macrophage cell line	[57]
Hyaluronic acid	After synthesis	rhombohedral	7	120	4	Antioxidant	Human fetal lung fibroblast cell line MRC5	[59]
		cubic	131.1 ± 0.7	-	-	Antioxidant	Chondrocytes	[62]
		spherical	4.15	100	10–20	Antioxidant	human umbilical cord mesenchymal stem cells (HucMSCs), in vivo	[65]
Chitosan	Before synthesis	-	5	-	-	Antioxidant Cytoprotective Anti-inflammatory	Human retinal pigment epithelial cell line ARPE-19	[108]
		spherical	100	-	40.9 ± 3.6	Antioxidant Regenerative	Ex vivo, in vivo	[115]
Chitosan	After synthesis	cubic	3−5	174 ± 1	9.6 ± 0.3	Biocompatibility Antioxidant	Human retinal pigment epithelial cell line ARPE-19	[77]
		microcubes	2500	-	-	Antioxidant. Antimicrobial Regenerative	*S. aureus*, *E. coli.* L929, in vivo	[78]
		spherical	-	-	3.61–24.40	Antimicrobial	*Escherichia coli*, *Bacillus subtilis*	[79]
		spherical	102, 112, 120	120	0.26	Antioxidant Antimicrobial Regenerative	*Staphylococcus aureus*, in vivo. L929	[83]
		granular	15–25	-	-	Regenerative	Adult rat spinal cord cell culture	[97]
Chitosan	Nanoparticles were not synthesized	-	40–50	-	-	Antioxidant Antimicrobial Regenerative	L929, MSSA, MRSA, in vivo	[90]
Collagen	After synthesis	-	-	-	-	Regenerative Antioxidant Anti-inflammatory	hDPSC, in vivo	[127]
		octahedron, rods, cubic	32.8, 16.4, 53.4	-	-	Antioxidant	Ovarian cancer cells	[128]
Gelatin	Before synthesis	-	43 ± 5	-	-	Antioxidant, antihypertrophic	Ex vivo. Cardiomyoblasts H9C2	[136]
Gelatin	After synthesis	spherical	50	-	-	Antioxidant Antimicrobial	*S. aureus*, *E. coli.* MC3T3-E1 cell line. In ovo	[84]
		spherical	35.5	110.01 ± 51.18	−12.35 ± 1.39	Antioxidant Regenerative	fibroblast-like human osteosarcoma cells MG-63	[133]
		-	22.13 ± 1.21	-	−19.1 ± 1.31	Regenerative	MC3T3-E1	[134]
		-	2.5–6.5	195 ± 3	22.4	Regenerative	NIH-3T3 fibroblast cells, in vivo	[140]
		-	11.6 ± 3.4	-	-	Regenerative	HaCaT keratinocytes, 3T3 fibroblasts, in vivo	[142]
		-	70	175.2 ± 6.9	−22.4 ± 0.8	Anti-inflammatory Regenerative	HaCaT, RAW264.7, in vivo	[144]
		-	-	-	-	Antioxidant Antimicrobial Regenerative	*S. aureus*, *E. coli*, in vivo	[147]
Gelatin	Nanoparticles were not synthesized	spherical	≤20	-	+18	Antimicrobial	*P. aeruginosa*	[146]
		-	<5	20	20	Antioxidant	Human neuroblastoma SH-SY5Y cells	[151]
		spherical	5–10	-	-	Regenerative	In vivo	[153]

**Table 2:** Result of interaction of carboxylic acid derivatives and nanoceria («-» means there are no data).

Excipient	Adding Excipient Before/After Synthesis CeO_2_	Morphology	Nanoparticle Size, nm	Hydrodynamic Diameter, nm	Zeta Potential, mV	Effects	Methods	Sources
Mellitic acid	Before synthesis	-	-	-	-	Stability	In vitro	[157]
Malic acid	After synthesis	-	4.2 ± 1.2	7.3	-	Stability	In vitro	[165]
Acetic acid	After synthesis	spherical	4.1			Antitumor	Human colorectal cancer cells (HT-29 cell line), human fetal foreskin fibroblast cell line (HFFF2 cell line)	[164]
N-acetylcysteine	Nanoparticles were not synthesized	-	20–30	--	-	Antioxidant	Human hepatocellular carcinoma cells SMMC-7721	[162]
Citrate	Before synthesis	isotropic	3–7	4.9	−20	Antioxidant	In vivo	[170]
		-	-	-	-	Regenerative Antioxidant	In vivo	[176]
		isotropic	2–5	4.9	−20	Antioxidant	In vivo	[186]
		-	3–4	60- 130	-	Regenerative	cell cultures of human fibroblasts, mesenchymal stem cells, and human keratinocytes	[17]
		-	3–5	60–120	-	Antimicrobial	*B. subtilis*, *B. cereus*, *S. aureus*, *P. aeruginosa*, *E. coli*, *P. vulgaris*, *C. albicans*, *A. brasielensis*	[178]
		-	-	-	-	Antimicrobial	*E. coli*, *Bacillus pyocyaneus*, *Staphylococcus aureus*, *Leuconostoc*, *Streptococcus faecalis*	[179]
		spherical	5	7	53 ± 7	Lack of pro- or antioxidant	In vivo	[180]
		-	5	-	−53 ± 7	Pro-oxidant	In vivo	[187]
		-	≤2	-	-	Cytoprotective	mouse fibroblasts (L929) and green monkey fibroblast-like cells (VERO)	[182]
		-	2.9	2.9 ± 0.3	−23.5	Antioxidant	In vivo	[184]
	After synthesis	faceted	2	9	-	High cellular uptake	NIH/3T3 mouse fibroblasts	[174]
		-	2	7	-	Toxicity in high doses	NIH/3T3 mouse fibroblasts	[175]
		polyhedral	5	8	−53 ± 7	Prooxidant	In vivo	[188]
		-	31.2	<200	−56 ± 8	Accumulation in the reticuloendothelial system	In vivo	[167]
		-	15–20	8–20	-	Regenerative	Fibroblasts, human mesenchymal stem cells, human keratinocytes	[17]
		-	1,7 ± 0,5	2.9	−23	Antioxidant	ex vivo RAW264.7 cells	[183]
		-	1–5	-	-	Antioxidant	in vivo	[171]
	Nanoparticles were not synthesized	pseudospherical	3 ± 1	7	−45 ± 5	Reduced toxicity	Caco-2 cells	[173]
		-	From 4.4 ± 0.9 to 22.9 ± 2.4	From 4.1 ± 0.1 to 6.2 ± 0.3	-	High aggregative stability	In vitro	[34]

**Table 3:** Result of interaction of fatty substances and nanoceria («-» means there are no data).

Excipient	Adding Excipient Before/After Synthesis CeO_2_	Morphology	Nanoparticle Size, nm	Hydrodynamic Diameter, nm	Zeta Potential, mV	Effects	Methods	Sources
Lecithin	After synthesis	-	-	-	-	Stability	Ram sperm	[193]
		-	5.82 ± 0.24	-	-	Antioxidant	keratinocyte cells, HaCaT line	[197]
Phosphatidylcholine	Before synthesis	-	3.7		From +10 to −38	Antioxidant	Murine insulinoma betaTC-tet cells	[194]

## Data Availability

No new data were created or analyzed in this study. Data sharing is not applicable to this article.
